# Graphene-based optical modulators

**DOI:** 10.1186/s11671-015-0866-7

**Published:** 2015-04-25

**Authors:** Siyuan Luo, Yanan Wang, Xin Tong, Zhiming Wang

**Affiliations:** Institute of Fundamental and Frontier Sciences, University of Electronic Science and Technology of China, Chengdu, 610054 People’s Republic of China; State Key Laboratory of Electronic Thin Film and Integrated Devices, School of Microelectronics and Solid-State Electronics, University of Electronic Science and Technology of China, Chengdu, 610054 People’s Republic of China

**Keywords:** Graphene, Optical modulator, Photonics, Absorption, Electro-absorption, All-optical, Terahertz, Broadband, Ultrafast

## Abstract

Optical modulators (OMs) are a key device in modern optical systems. Due to its unique optical properties, graphene has been recently utilized in the fabrication of optical modulators, which promise high performance such as broadband response, high modulation speed, and high modulation depth. In this paper, the latest experimental and theoretical demonstrations of graphene optical modulators (GOMs) with different structures and functions are reviewed. Particularly, the principles of electro-optical and all-optical modulators are illustrated. Additionally, the limitation of GOMs and possible methods to improve performance and practicability are discussed. At last, graphene terahertz modulators (GTMs) are introduced.

## Review

### Introduction

As one of the key components in photonics systems, an optical modulator is a device used to control the fundamental characteristics of a carrier light propagating in free space or in an optical waveguide upon an external electronics/photonics signal [1]. In order to meet specific requirements in applications, such as modern lasers, optical communication, and terahertz communication, various designs have been demonstrated. And thanks to the latest development in nanotechnology and material science, advanced-function materials are progressively involved in device fabrication. For instance, group III-V materials [2], germanium [3,4], polymers [5,6], and graphene [7,8] have been applied and incorporated to silicon-based modulators to form hybrid devices, with the aim to improve the modulation speed, broaden the modulation range, and reduce the device footprint and energy consumption. According to the parameters being modulated, these devices can be categorized as amplitude, phase, or polarization modulators. Generally, amplitude modulation is the most common due to its classified system. And the performance can be characterized by optical bandwidth, modulation depth, modulation speed, insertion loss, area efficiency (footprint), and power consumption [9].

As the prime material for the semiconductor industry, silicon modulators have to be fabricated in large scale to obtain enough modulation depth, due to a relatively weak high-order electro-optical effect. On the other hand, modulators based on germanium and other compounds have problems to be integrated with current complementary metal-oxide-semiconductor (CMOS) techniques. For modulators with resonators, narrow modulation bandwidth limits their development. By contrast, graphene can cover the needs of scale, speed, and techniques. And integration with graphene can help current modulators to improve their performance.

Graphene, a single layer of hexagonally packed carbon atoms, was first isolated from graphite via mechanical exfoliation in 2004. For these highly confined two-dimensional crystals, in-plane carbon atoms are connected by strong σ-bonds, while adjacent layers only share weak van de Waals force. The unique crystalline structure endows graphene extraordinary electronic, optical, thermal, and mechanical properties. Graphene is expected to grow into the new silicon in future electronics and photonics. Many proof-of-concept photonics devices based on graphene, including photodetectors [10,11], ultrafast lasers [12,13], polarization controllers [14], and plasmonic structures [15-17], have been demonstrated.

**Figure 1:**
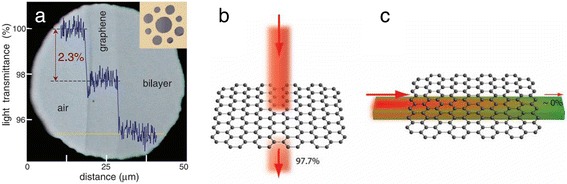
Optical absorption in graphene. **(a)** Optical absorption of approximately 2.3% for pristine graphene from ref. [13]. (Inset) Graphene crystallites were placed over a metal with several apertures. **(b)** For vertical incident light, an optical absorption of approximately 2.3% can be achieved by single-layer graphene. **(c)** By integrating graphene along with a waveguide (i.e., light transmits horizontally through graphene), large light-graphene interaction and higher absorption can be achieved. Reproduced from ref. [14].

For applications in optical modulators, graphene has its unique advantages as follows: (1) High modulation speed: With a carrier mobility as high as 200,000 cm^2^/(V · s) at room temperature, graphene is considered as one of the fundaments of next-generation ultrafast electronics/photonics devices [18]. Ultrafast (picoseconds) processes in graphene, such as photocarrier generation and relaxation, offer graphene a possibility to operate at over hundreds of GHz [19]. Thus, the Fermi level, which is directly related to the optical absorption of graphene, can be rapidly modulated through gating voltage doping. (2) Wide optical bandwidth: Due to its unique electronic structure [10,11], graphene has a constant absorption of *πα* = 2.293% from visible to infrared wavelengths [12], where *α = e*^2^/*hc* denotes the fine-structure constant [13], as is shown in Figure 1a. This bandwidth covers the optical fiber communication bandwidth, typically from 1,300 to 1,600 nm. (3) High optical absorption: Considering only one atom thickness, an optical absorption of approximately 2.3%, which is approximately 50 times higher than that of GaAs of the same thickness, is quite high. By integrating graphene along with a waveguide, the light-graphene interaction length can be further improved [14], as shown in Figure 1b,c. In this original structure, an absorption (modulation depth per unit length) of 0.2 ~ 1 dB/μm can be achieved. Higher absorption will help to reduce the scale of the device (footprint). (4) CMOS-compatible: During the past decade, large-scale graphene can be integrated using CMOS-compatible processes [15,16]. Moreover, because of Pauli blocking (band filling), saturable absorption has been observed [17,20], which makes it possible to fabricate all-optical graphene optical modulators.

In this review article, we provide a brief overview of graphene-based optical modulators. Our survey is not intended to cover every single device reported in prior publication, but rather to introduce some typical designs and highlight some recent notable work. Classified by whether electrical elements are involved or not, the principle and paradigms of electro-optical and all-optical graphene optical modulators are elaborated in the ‘Electro-optical graphene optical modulator’ and ‘All-optical grapheme optical modulator’ sections, respectively. In addition, graphene-based material systems for THz wave modulation are discussed in the ‘Graphene terahertz modulator’ section. The article closes with a final conclusion and outlook in the ‘Conclusions’ section.

### Electro-optical graphene optical modulator

#### Mechanism of electro-absorption

Due to the sp^2^ hybridization of carbon atoms, graphene has a unique electronic structure in that the conduction band and valence band meet at Dirac points like two cones [10,11]. A linear energy-momentum dispersion relation can be noted in the vicinity of Dirac points and carries behavior that can be modeled as massless Dirac fermions.

**Figure 2:**
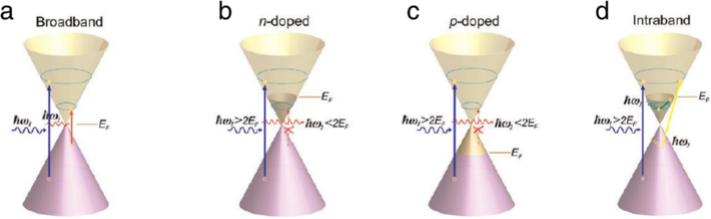
Possible optical transitions in graphene. **(a)** Optical transition in pristine graphene. **(b)** Optical transition in n-doped (or driven by large positive voltage) graphene. **(c)** Optical transition in p-doped (or driven by large negative voltage) graphene. **(d)** Intraband transition in graphene. Reproduced from ref. [1].

For pristine graphene, electrons can be excited by incident photons with a broad range of energies and only interband transition is permitted (Figure 2a). As a consequence of universal optical conductance, the transmittance of pristine graphene is frequency-independent and only determined by the fine-structure constant *α = e*^*2*^/*ħc* (where *e* is the electronic charge, *ħ* is Planck’s constant divided by 2*π*, and *c* is the velocity of light) [13]:

1 $$ T={\left(1+2\pi G/c\right)}^{-2}\approx 1-\pi \alpha \approx 0.977 $$

While sufficiently doped, the optical transition of graphene is mainly determined by chemical potential *μ* (Fermi level *E*_F_), which can be controlled by chemical doping or electrical gating. The Kubo formula can be used to describe the dynamic response of graphene, including interband transition and intraband transition [1]:

2 $$ \sigma ={\sigma}_{\mathrm{intra}}+{\sigma}_{\mathrm{inter}}'+i{\sigma}_{\mathrm{inter}}" $$

3 $$ {\sigma}_{\mathrm{intra}}={\sigma}_0\frac{4\mu }{\pi}\frac{1}{\hslash {\tau}_1-i\hslash \omega } $$

4 $$ {\sigma}_{\mathrm{inter}}'={\sigma}_0\left(1+\frac{1}{\pi } \arctan \frac{\hslash \omega -2\mu }{\hslash {\tau}_2}-\frac{1}{\pi } \arctan \frac{\hslash \omega +2\mu }{\hslash {\tau}_2}\right) $$

5 $$ {\sigma}_{\mathrm{inter}}"=-{\sigma}_0\frac{1}{2\pi } \ln \frac{{\left(\hslash \omega +2\mu \right)}^2+{\hslash}^2{\tau_2}^2}{{\left(\hslash \omega -2\mu \right)}^2+{\hslash}^2{\tau_2}^2} $$

Both interband transition and intraband transition are related to chemical potential *μ* and the frequency of incident light *ω*. When *μ =* 0, no intraband transition will happen. When |*μ*| < *ħ ω*/2, (slightly n-doped or p-doped) optical transition is dominated by interband transition. In n- and p-doped (corresponding to positive and negative gating voltage) graphene, the incident photons with energy less than 2*E*_F_ cannot be absorbed. This is because the electron states in the conduction band are filled up as shown in Figure 2b or there are no electrons in the valence band available for interband transition as shown in Figure 2c. Thus, if the incident light is fixed, by electrically tuning the Fermi level, interband transitions can be turned on and off [21,22]. When |*μ*| < *ħ ω*/2, the intraband transition related to the terahertz range will be dominant [23-25]. At this condition, plasmon momentum enhancement is allowed and propagation of surface plasmon in graphene becomes possible [26-28].

In earlier theory demonstrations, graphene was treated as an isotropic material [29,30]. Graphene can transfer from dielectric-like to metallic-like when the permittivity is tuned to approach zero. Recently, graphene became well accepted as an anisotropic material. When graphene was treated as an anisotropic material [31,32], a linear relation between its in-plane permittivity and effective mode index can be observed. The electric distributions are also different in or out of graphene when it is treated as an isotropic or anisotropic material [33]. In this case, the in-plane permittivity can be tuned by the chemical potential, while the out-of-plane permittivity (in a direction perpendicular to the graphene sheet) does not [33].

#### Basic designs of electro-optical graphene optical modulator (GOM)

**Figure 3:**
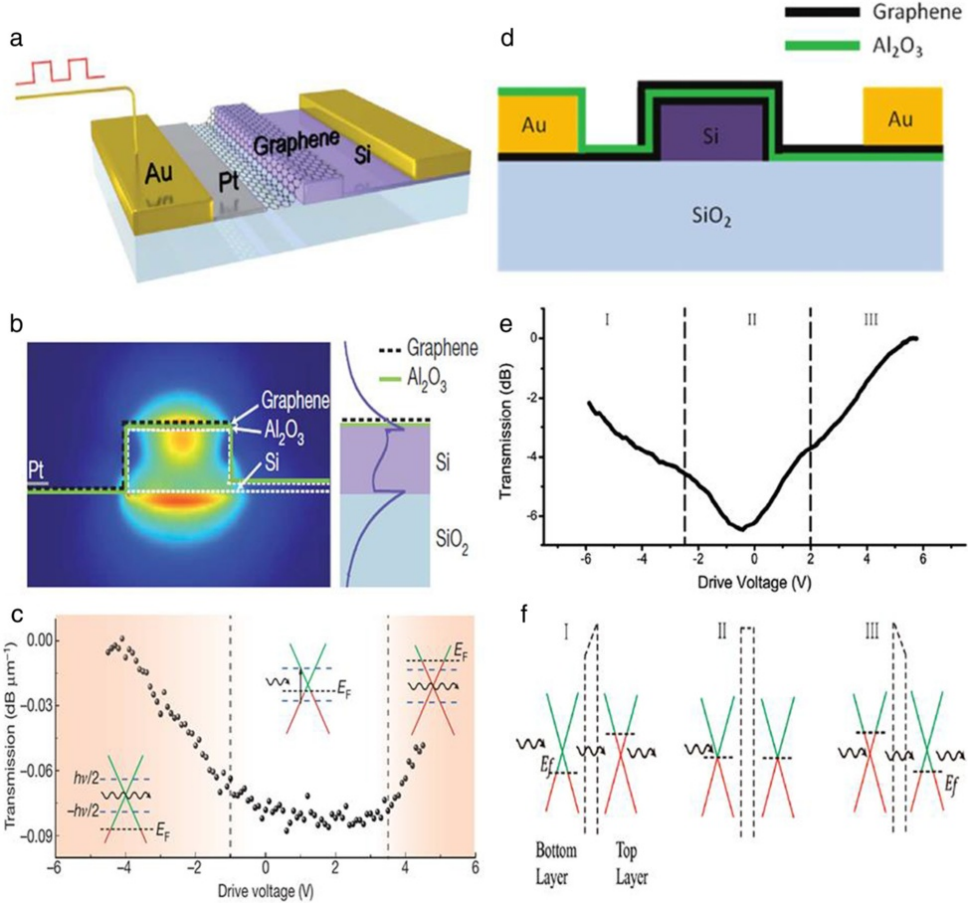
Single-layer and double-layer GOMs. **(a)** Schematic of a single-layer GOM. The graphene film is separated from the silicon waveguide by a thin Al_2_O_3_ layer which is not shown. Pt- and Si-doped layers are deposited to connect graphene and gold electrode. The Si waveguide is also shallowly doped with boron to reduce the cascade resistance. **(b)** Cross section of a single-layer GOM. Left: cross section of the device with optical field distribution. Right: electric field distribution in the waveguide. **(c)** Transmission at different gating voltages in a single-layer GOM. When the Fermi level is close to the Dirac point, optical absorption occurs and transmission reduces. When large gating voltages are applied, optical absorption blocks and transmission increases. Reproduced from ref. [7]. **(d)** Schematic of a double-layer GOM. The two graphene layers are separated by a thin film of Al_2_O_3_. And the bottom graphene layer directly contacts the Si waveguide. **(e)** Transmission of carrier light at different gating voltages, which is similar to that of the single-layer GOM. **(f)** Tuned Fermi level and optical absorption behavior in double-layer graphene. Even though the Fermi levels are different when large gating voltages are applied, both layers tend to be transparent. When the Fermi levels are close to the Dirac point, both layers absorb the incident light, which results in a higher modulation depth. Reproduced from ref. [8].

In 2011, Liu et al. first experimentally demonstrated a GOM by integrating a monolayer graphene sheet on a Si waveguide as shown in Figure 3a [7]. The waveguide propagates light and graphene will offer absorption. The field distribution of the propagating light is shown in Figure 3b, which is very important to modulation depth. External gating voltage was used to control the Fermi level of graphene and resulted in changes of transmission in the Si waveguide as shown in Figure 3c. This original modulator can work at a broad bandwidth from 1,350 to 1,600 nm, which covers the wavelength of the optical fiber communication system we are using today. A modulation depth per unit length of 0.1 dB/μm was achieved, and the footprint of this modulator was 25 μm^2^. Right after the first demonstration, they further improved the modulator by integrating double-layer graphene on the top of a Si waveguide [8], as is shown in Figure 3d. This double-layer GOM has a similar transmission property to that of the single-layer GOM in their former work, as is shown in Figure 3e. The two graphene layers with a p-oxide-n-like structure are simultaneously absorptive or transparent for incident light, as is shown in Figure 3f. As is expected, a higher modulation depth of 0.16 dB/μm was observed due to the double-layer graphene, which implies a smaller footprint at 3-dB modulation. This performance is comparable to that of traditional optical modulators made of Si [9] and GeSi [34].

#### Advanced structures for electro-optical GOM

**Figure 4:**
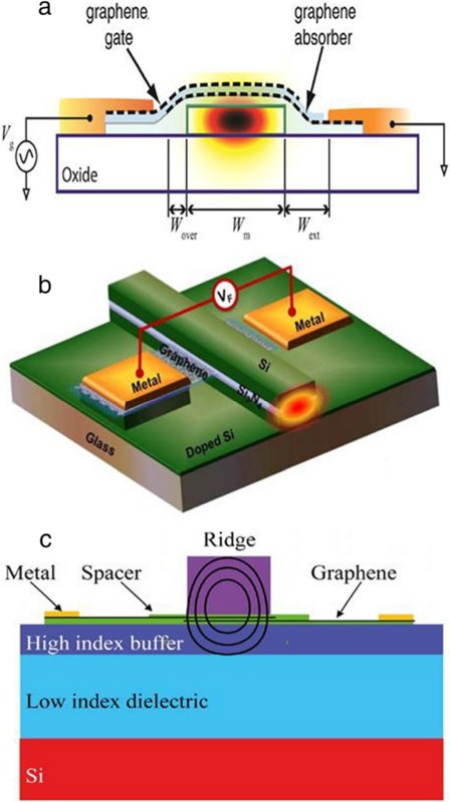
GOM with double-layer graphene at different positions. **(a)** On the top of the waveguide (reproduced from ref. [35]); **(b)** in the center of the waveguide (reproduced from ref. [30]); **(c)** at the bottom of the ridge of the rib waveguide (reproduced from ref. [36]).

With the aim to achieve higher performance, different structures have been developed. High modulation depth not only brings a higher signal quality but also helps to reduce the footprint. Simply increasing the peak-to-peak gate voltage swing can achieve high modulation depth at the expense of increased power consumption. Improving the graphene-light interaction can fundamentally increase the performance. Similar to Liu et al.’s work, Koester and Li simulated a graphene-on-silicon structure [35] as shown in Figure 4a. Although a modulation speed of 120 GHz is possible, the interaction length is 60 μm when achieving a 3-dB modulation (corresponding to 0.05 dB/μm). Lu and Zhao theoretically showed that graphene sheets should be placed at the maximum of the electric field [30]. They designed a structure in which the graphene sheet is sandwiched in the center of the waveguide as shown in Figure 4b. A modulation depth of 3.75 dB/μm was achieved, which is much higher than that of the graphene-on-silicon structure. However, placing the graphene sheet in the waveguide is difficult to be realized. Imperfect fabrication such as mismatch of the upper part and bottom part of the waveguide may influence the signal quality. Gosciniak and Tan theoretically proposed a method to avoid the technique challenges and at the same time placed graphene sheets close to the maximum of the electric field [36], as is shown in Figure 4c. A Si rib waveguide was deposited on the substrate covering the double-layer graphene sheet. The Si waveguide was specially designed in size to form an egg-like field distribution which is represented by the black line in Figure 4c. The double-layer graphene was separated by a thin dielectric spacer forming a parallel capacitor model. Modulation depths of 5.05 dB/μm for TM mode and 0.29 dB/μm for TE mode were achieved. With this high modulation depth, nanoscale devices with 3-dB modulation depth are possible. It should be noted that in this structure, part of the mode was pushed into the buffer layer, which may make the mode field weaker. Thus, balance designation is necessary. Without waveguides, transmission and reflection structures provide different applications. Lee et al. fabricated a reflection GOM within sub-wavelength thickness [37]. Later, by improving graphene supercapacitors, Polat and Kocabas achieved broadband GOMs and compared the performance of transmission and reflection [38]. As is expected, the reflection structure showed a higher modulation depth. In the aspect of insertion loss, an insertion loss of 3.3 dB with a modulation of 16 dB was experimentally demonstrated, recently [39].

#### Integration of graphene with other optical modulators

**Figure 5:**
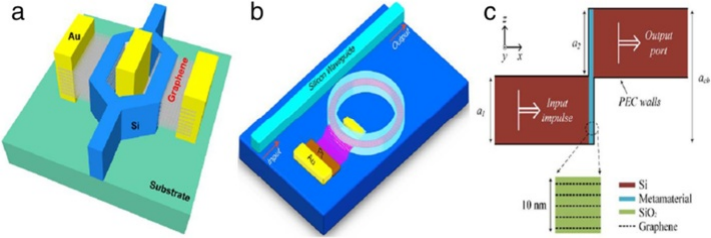
Graphene-enhanced optical modulator. **(a)** Mach-Zehnder modulator with eight-layer graphene integrated in the arms. Reproduced from ref. [36]. **(b)** Ring modulator with graphene embedded in the ring resonator. Reproduced from ref. [41]. **(c)** Metamaterial channel with multi-layer graphene embedded in the resonator. Reproduced from ref. [42].

By integrating graphene, the performance of current optical modulators can be further enhanced. Hao et al. theoretically demonstrated a Mach-Zehnder modulator with eight-layer graphene embedded [31], as is shown in Figure 5a. The embedded graphene sheets significantly enhanced the electro-refraction, which is helpful to reduce the footprint to 4 × 30 μm^2^ and modulation arm length of 27.57 μm in the Mach-Zehnder modulator. And they further reduced the modulation arm length to 16.5 μm [32]. Moreover, graphene also helps to reduce the chirp in the Mach-Zehnder modulator [29]. The graphene-embedded design also benefits the ring modulator. An optical modulator based on the critical coupling concept [40] can be realized when assisted by graphene [41]. With the driving voltage lower than 1.2 V, this modulator was compatible with low-voltage CMOS technology. Recently, Du et al. demonstrated a ring modulator with a shift rate of 1.08 nm/V at resonance peak, which is two orders of magnitude higher than that of current ring modulators [42], as is shown in Figure 5b. By simulating a graphene-silica permittivity-tunable metamaterial, a GOM with a footprint of 0.01 μm^2^ was reported recently [43], as is shown in Figure 5c.

In addition, a device integrating both GOMs and a graphene optical photodetector was experimentally demonstrated [44]. Recently, Zhou et al. first theoretically found a quasilinear relation between the phase change and chemical potential of graphene, which implied an optical phase modulator [45].

#### RC constant limit in electro-optical GOM

In theory, the high carrier mobility of graphene will lead to an ultrahigh modulation speed. However, in experimental demonstration, the modulation speed is still limited at approximately 1 GHz [7,8] lower [39] in electro-optical GOMs. The reason is the ‘electrical bottleneck’ - RC constant. The electronic circuit of this device can be equivalent to RC low-pass filter (LPF). The 3 dB cut-off frequency of electronic signal can be calculated by *f = 1*/*2πRC*, where *R* is the total cascade resistance and *C* is the total capacitance between counter electrodes. These factors can be measured by a network analyzer. The all-optical method is an efficient way to avoid this bottleneck.

### All-optical graphene optical modulator

The future optical fiber communication system requires a modulator whose operation speed is larger than 100 Ghz [46]. Although the graphene-based modulator has the potential to obtain a modulation rate of 500 GHz, the practical electro-absorption modulator based on graphene is limited to approximately 1 GHz due to the RC constant [7,8]. A direct method to avoid this ‘electrical bottleneck’ is to make the modulator all-optical. That is, light modulates light. The all-optical graphene optical modulators demonstrated at present are based on saturable absorption in graphene.

#### Mechanism of saturable absorption

Saturable absorption is a property of materials where the absorption of light is decreased to a steady level at sufficiently high incident light intensity [1]. This optical nonlinearity is widely applied to generate short laser pulses as optical absorber in mode-locked lasers [20,47]. It is worth noting that high incident optical intensity may damage the material during absorption. Although many semiconductors such as GaAs also show saturable absorption, only those whose saturable intensity is much lower than the optical damage threshold can be used in practical devices [48]. Optical devices based on graphene with high optical damage threshold have been fabricated [49]. Moreover, in saturable absorption devices, compared with single-walled carbon nanotubes (SWNTs) [17] or semiconductor saturable absorber mirrors (SESAMs) [50], graphene is much easier to be fabricated without band gap engineering or chirality (diameter) control.

**Figure 6:**
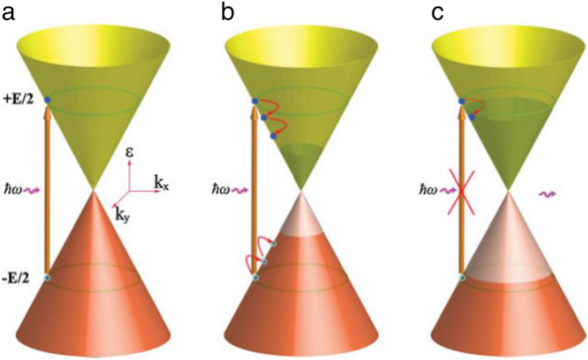
Processes of saturable absorption in graphene. **(a)** Optical interband transition excited by incident light. **(b)** The photogenerated carriers redistribute a Fermi-Dirac distribution. **(c)** Further absorption is blocked under sufficient intensity of incident light. Reproduced from ref. [50].

**Figure 7:**
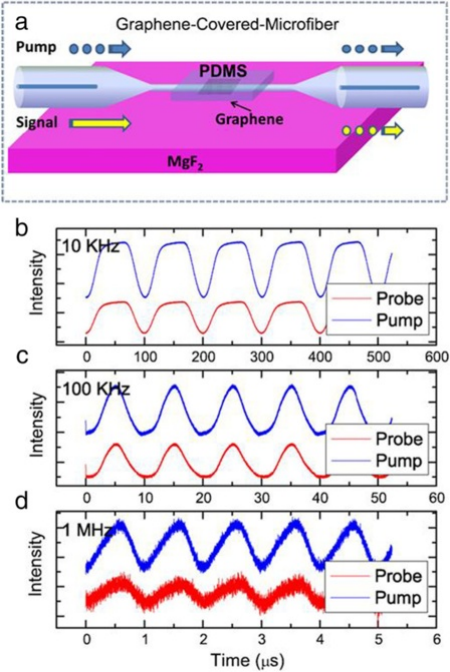
Schematic of a graphene-covered microfiber structure and modulation result. **(a)** Schematic structure of a graphene-covered microfiber. A PDMS-supported graphene covered on the microfiber. Pump light and carrier light (signal) propagate in the waveguide simultaneously and the intensity of carrier light follow the changes of pump light with a modulation speed of **(b)** 10 kHz, **(c)** 100 kHz, and **(d)** 1 MHz. Reproduced from ref. [51].

The schematic saturable absorption process is shown in Figure 6 [51]. Excited by pump light, optical interband transition occurs as shown in Figure 6a. Graphene absorbs incident light regardless of wavelength. Then the thermalized photogenerated carriers will cool down and redistribute a Fermi-Dirac distribution. Electron–hole recombination and intraband phonon scattering accompany this redistribution as shown in Figure 6b. With sufficient intensity of pump light, the conduction band and valence band will be filled up by electrons and holes, respectively. Thus, due to Pauli blocking (no two electrons can fill the same state), further absorption is blocked, achieving saturable absorption or absorption blenching as is shown in Figure 6c. Above all, in this circumstance, other light whose energy is less than the pump light will not be absorbed by graphene. When pump light (high energy) and carrier light (low energy) simultaneously transmit through graphene, sufficiently increasing the intensity of pump light can limit the absorption of carrier light. As a result, as is shown in Figure 7b,c,d, the intensity of carrier light will follow that of pump light, which implies all-optical modulation.

#### Basic designs of all-optical GOM

Liu et al. firstly experimentally showed all-optical modulation using a graphene-covered microfiber, which is compatible with the optical fiber system [52]. A chemical vapor deposition (CVD)-synthesized graphene film is dry transferred by polydimethylsiloxane (PDMS) to cover the microfiber on MgF_2_ substrate, as is shown in Figure 7a. In the substrate-supported structure, the substrate should have a low refractive index to guarantee the total reflection. Pump light (1,060 nm) and carrier (signal) light (1,550 nm) together transmit through the microfiber and the intensity of carrier light varied with pump light, as is shown in Figure 7b,c,d. In this work, a modulation speed of only 1 MHz is achieved due to the low switching frequency of pump light. A modulation depth of approximately 5 dB is achieved by single-layer graphene. And as is expected, a higher modulation depth of approximately 13 dB is achieved by double-layer graphene.

#### Advanced structures for all-optical GOM

**Figure 8:**
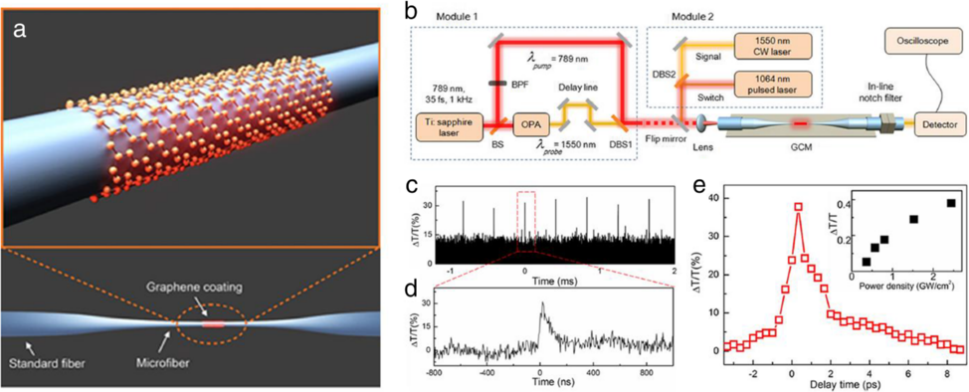
Schematic illustration and ultrafast property of all-optical graphene optical modulator. **(a)** Graphene-clad microfiber (GCM) structure. **(b)** Schematic illustration of measurement. Module 1: light source for ultrafast measurement. This module simultaneously outputs 789-nm pulses and 1,550-nm pulses by transforming a 789-nm femtosecond laser source. A delay line is used to adjust the delay between 789-nm pulse and 1,550-nm pulse. Module 2: light source for modulation. **(c)** 1,550-nm carrier light modulated by a 5-ns 1,064-nm pump light pulse train. The light sources are in Module 2. **(d)** Time profile of switched-out pulse. Each modulated pulse has an approximately 80-ns tail owing to the slow recovery time of the photodetector. **(e)** Measurement of response time showing approximately 2.2 ps. The inset shows the dependence of modulation depth on pump intensity. Reproduced from ref. [52].

Actually, the all-optical graphene modulator is able to reach ultrafast modulation speed without RC limitation. A practical ultrafast all-optical graphene optical modulator, which is compatible with the current high-speed fiber optical communication system, was fabricated by Li et al. [53]. The structure they used is a graphene-clad microfiber (GCM), as is shown in Figure 8a, which has been reported for mode-locked fiber lasers [54,55]. When 1,060-nm pump laser pulses (approximately 5 ns, 24 kHz) and 1,550-nm CW light were coupled to the GCM (Figure 8b, Module 2), it was found that the photodetector cannot follow due to the slow recovery time. As is shown in Figure 8c,d, the long tail (approximately 80 ns) may mistake the ultrafast measurement of modulation speed. Considering this, successively releasing femtosecond pump light and carrier light by using a delay line (Figure 8b, Module 1) and detecting the intensity of carrier light can measure the response of saturable absorption. This absorption has an ultrafast excitation and approximately 2.2 ps decay time as shown in Figure 8e. The decay time include the relaxation time of carrier-carrier scattering (tens to hundreds of femtoseconds) and that of carrier-phonon scattering (approximately 1 to a few picoseconds) [56-58]. This ultrafast response time implies a potential to achieve a modulation speed of approximately 200 GHz for Gaussian pulses. Finally, a modulation depth of 38% was achieved within 30-μm-long graphene.

Theoretically, if the intensity of pump light is strong enough (lower than the optical damage threshold), graphene can be totally transparent to carrier light. Thus, the maximum modulation depth is determined by the optical absorption when the pump light is off, which is largely related to the interaction length and position of the graphene sheet. However, in the works above, sufficiently saturable absorption is not achieved and absorption of carrier light is gradually varied with the changes of pump intensity as shown in the inset of Figure 8e [53]. In the aspect of transmission property, graphene integrated with a microfiber has higher absorption along with increasing wavelength, which can be explained by higher evanescent field for longer wavelength at the graphene interface [59]. In addition, different polarization of the pump light can result in approximately 1 dB change of modulation depth [55].

### Graphene terahertz modulator (GTM)

In the past decades, terahertz (THz) technology was found to be applied in diverse areas such as astronomy, biology/medicine [60], communications [61], and defense [62]. Although numerous advances have been achieved, most of them focus on emitters and detectors. Devices like active filters and modulators which can be integrated with current solid-state continuous-wave (CW) terahertz sources and detectors such as quantum cascade lasers [63], resonant tunneling diode oscillators [64], Schottky diodes [65], backward diodes [66], or future graphene-based terahertz devices [67] still need to be improved [68]. As a gapless semiconductor, graphene is a natural material for long-wave applications such as THz. With the advantages mentioned in the introduction, graphene shows great potential in modulators and detectors [67].

The optical conductivity of graphene is determined by interband transition and intraband transition, respectively, mainly for short wavelength (infrared and visible) and long wavelength (terahertz) [23-25]. Thus, electrostatically tuning the density of states (DOS) available for intraband transitions provides the possibility to effectively control the terahertz absorption [69,70]. As a result, large gating voltage is usually used. A high modulation depth of >90% has been shown by employing graphene in place of a metal gate in an AlGaAs/GaAs two-dimensional electron-gas (2DEG) terahertz modulator, which provides a modulation of <30 only [70].

#### Electro-optical GTM

**Figure 9:**
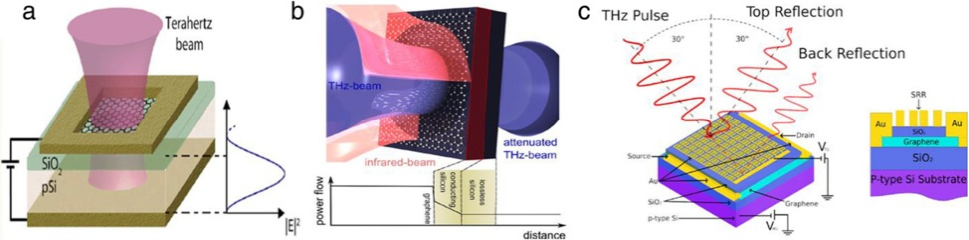
GTMs. **(a)** Electro-optical GTM. Gating voltage is applied between the top electrode and back electrode (also used to reflect the terahertz beam) to modulate the terahertz absorption of graphene. The line plot shows the electric field in the substrate. The optical thickness provides a critical influence on the electric field in the substrate and graphene. Reproduced from ref. [71]. **(b)** All-optical GTM. Incident infrared beam modulates the terahertz absorption of graphene. The inset shows the power flow in the substrate. The conducting silicon layer also contributes to attenuate the terahertz beam. Reproduced from ref. [73]. **(c)** GTM with split-ring resonators (SRRs). Right: the structure of SRR GTM. A SiO_2_ was deposited to separate the graphene sheet and metal SRRs defined by e-beam lithography. Left: the reflected power of terahertz from the top and back sample surfaces at different bias VTG of the split-ring arrays with respect to graphene can be measured. Reproduced from ref. [79].

In 2012, Sensale-Rodriguez et al. first experimentally demonstrated a GTM enabled only by intraband transitions [71]. Later on, they successfully used an electro-absorption GTM to control the reflectance of the terahertz wave [72]. The reflection structure they used is shown in Figure 9a. When the Fermi level in graphene is tuned to the Dirac point, intraband transition is blocked. Thus, absorption is at its minima and the reflectance of the device is at its maxima. On the other hand, when the Fermi level shifts into the valence or conduction band of graphene, the increase of density of states available for intraband transitions leads to a higher absorption. It should be noted that if a reflection structure is used, the optical thickness of the substrate needs to be well controlled. When the substrate optical thickness is an odd-multiple of a quarter-wavelength, the electric field in graphene is maximized and absorption can be deeply modulated. On the contrary, when the substrate optical thickness is an even-multiple of a quarter-wavelength, the electric field in graphene disappears and absorption does not occur. As a result, a modulation depth of 64% and a low insertion loss of approximately 2 dB are achieved. Recently, they experimentally applied arrays of electro-absorption GTMs as electrically reconfigurable patterns for terahertz cameras [73]. A similar structure was also adopted by Lee et al. to fabricate modulators for IR range within sub-wavelength thickness [37].

#### All-optical GTM

Following Sensale-Rodriguez et al.’s first demonstration of GTM [71], Weis et al. fabricated an all-optical GTM in the same year, 2012 [74]. They deposited graphene on silicon (GOS) to enhance the absorption as shown in Figure 9b. Upon infrared photodoping, a broadband modulation from 0.2 to 2 THz was achieved. Moreover, the modulator showed a maximum modulation depth of 99%.

#### GTM with resonators

Due to accurate and deep modulation in the THz range, integration with resonators shows a way to cover special needs [75-77]. In graphene-integrated modulators, the resonators not only enhance the interaction between graphene and terahertz wave but also bring the advantage to decrease the bias [78]. Degl’Innocenti et al. recently integrated metallic split-ring resonators (SRRs) and single-layer graphene on one substrate [79]. A modulation depth of 18% and a bandwidth from 2.2 to 3.1 THz were achieved. Additionally, the structure, as is shown in Figure 9c, showed a low bias of 0.5 V [80]. Recently, using resonators, terahertz modulators based on metamaterial and graphene have also been studied [81]. However, complex design and fabrication increase the difficulty and cost.

## Conclusions

Optical modulators are an important device to the current and future optical systems and still need to be improved. Graphene shows great potential in fabricating broadband and ultrafast optical modulators. Optical transition including interband and intraband transitions in graphene is the main process during absorption. Electro-optical GOMs have been demonstrated while the modulation speed is limited to approximately 1 GHz due to the RC constant. The position of the graphene sheet efficiently influences the light-graphene interaction. Higher modulation depth can be easily achieved by placing graphene close to the maximum of the electric field. Following the first demonstration, many optical modulators enhanced by graphene have been theoretically and experimentally demonstrated. However, higher modulation speed is necessary for current electro-optical GOMs. Driven by saturable absorption, all-optical GOMs show a potential of ultrafast modulation speed due to the ultrafast relaxation time. But direct measurement of ultrafast modulation has not been demonstrated. In the field of terahertz, graphene has a prominent advantage of high modulation depth. Electro-optical and all-optical modulation are both possible. In principle, theoretical simulations go much further than experiment. GOMs with new structures and high performance tend to be demonstrated in the near future.
